# Maternal Downward Neighborhood Income Mobility and Newborn Discharge to Child Protective Services

**DOI:** 10.1001/jamanetworkopen.2024.40604

**Published:** 2024-10-23

**Authors:** Jennifer A. Jairam, Eyal Cohen, Christina Diong, Howard Berger, Jun Guan, Emily Ana Butler, Joel G. Ray

**Affiliations:** 1 Department of Medicine Research, St Michael’s Hospital, Toronto, Ontario, Canada; 2 ICES, Toronto, Ontario, Canada; 3 Child Health Evaluative Sciences, SickKids Research Institute, Toronto, Ontario, Canada; 4 Department of Pediatrics, The Hospital for Sick Children, Toronto, Ontario, Canada; 5 Edwin S. H. Leong Centre for Healthy Children, University of Toronto, Toronto, Ontario, Canada; 6 Department of Medicine, St Michael’s Hospital, University of Toronto, Toronto, Ontario, Canada; 7 Department of Obstetrics and Gynaecology, St Michael’s Hospital, Toronto, Ontario, Canada; 8 Keenan Research Centre, St Michael’s Hospital, Toronto, Ontario, Canada

## Abstract

This cohort study examines whether there is an association between a mother moving into a neighborhood with lower income between births and newborn custody by child protective services at birth.

## Introduction

Separation at birth can be detrimental to both mother and infant.^1^ Maintaining their union can have physiological and neurodevelopmental benefits for both.^2,3^ However, if a mother is deemed unable to care for her newborn, then the child may be assigned to child protective services.^1^

Limited research suggests that there is a disproportionate burden of newborn separation by child welfare services among mothers residing in lower-income or urban neighborhoods and those who frequently change their residence.^1,4^ Longitudinal data are lacking about whether downward neighborhood income mobility between 2 births is associated with newborn custody by child protective services.

## Methods

This population-based cohort study used multiple linked administrative databases from Ontario, Canada, within a universal health care system. Included were all female individuals with at least 2 consecutive singleton in-hospital births at 20 to 42 weeks’ gestation between April 1, 2002, and March 31, 2018, who were residing in a neighborhood with an income quintile (Q) of 2, 3, 4, or 5 (highest) at the time of the first birth (eTable 1 in Supplement 1).

Data use was authorized under section 45 of Ontario’s *Personal Health Information Protection Act* and exempt from a research ethics board review and informed consent. This study followed the STROBE reporting guidelines.

The study exposure, degree of downward neighborhood income mobility between the first and second births, was categorized as downward movement by: (1) 1 Q, (2) 2 Qs, (3) 3 Qs, or (4) 4 Qs, each relative to (5) no downward income mobility, defined as residing in the same income Q at both births or moving to a neighborhood with a higher income Q between births. The study outcome, newborn discharge to child protective services during the second birth hospitalization, up to the discharge date, was based on a discharge disposition variable in the newborn’s hospital record at the second birth. This approach was validated elsewhere.^5^ Study variables and databases are detailed in eTables 1 and 2 in Supplement 1.

We generated relative risks (RRs) and 95% CIs for newborn discharge to child protective services at the second birth by degree of maternal downward neighborhood income mobility. The eMethods in Supplement 1 details all analyses. Statistical analyses were conducted from March to August 2024 using SAS version 9.4 (SAS Institute). The magnitude of RRs and precision of corresponding 95% CIs indicated significant differences between exposure groups.

## Results

Within the cohort of 433 575 women, 104 663 (24.1%) experienced downward neighborhood income mobility between births (mean [SD] age, 31.0 [5.1] years), and 328 912 (75.9%) did not (mean [SD] age, 32.3 [4.5] years) (Table). Those with downward income mobility were younger and had a longer median time interval between births (Table). Relative to mothers with no downward neighborhood income mobility, the adjusted RRs for newborn discharge to child protective services at the second birth were 2.34 (95% CI, 1.92-2.85) for those with downward income mobility by 1 Q, 4.22 (95% CI, 3.22-5.53) by 2 Qs, 6.12 (95% CI, 4.23-8.83) by 3 Qs, and 7.02 (95% CI, 4.02-12.26) by 4 Qs (Figure).

**Table.** Maternal and Newborn Characteristics Among Those Who Did and Did Not Experience Downward Mobility in Neighborhood Income Q Between 2 Consecutive Births in Ontario, Canada, 2002 to 2018

Characteristic	Participants, No. (%)		Standardized difference^a^
	Any downward income mobility between births (n = 104 663)	No downward income mobility between births (n = 328 912)	
**Maternal, at the first birth**			
Neighborhood income Q			
2	16 949 (16.2)	93 891 (28.5)	0.30
3	24 700 (23.6)	89 517 (27.2)	0.08
4	31 547 (30.1)	86 155 (26.2)	0.09
5 (Highest)	31 467 (30.1)	59 349 (18.0)	0.28
**Maternal, 1 to 365 d prior the second birth**			
No. of comorbidities^b^			
≤2	95 471 (91.2)	310 749 (94.5)	0.13
3-4	6797 (6.5)	14 405 (4.4)	0.09
5-6	1734 (1.7)	2897 (0.9)	0.07
≥ 7	661 (0.6)	861 (0.3)	0.06
**Maternal, at the second birth**			
Age, y			
Mean (SD)	31.0 (5.1)	32.3 (4.5)	0.27
16-24	12 123 (11.6)	16 556 (5.0)	0.24
25-29	27 049 (25.8)	65 818 (20.0)	0.14
30-50	65 491 (62.6)	246 538 (75.0)	0.27
Time interval between births, mo			
Median (IQR)	36 (26-53)	30 (23-42)	0.32
6-17	6187 (5.9)	26 401 (8.0)	0.08
18-60	79 517 (76.0)	271 349 (82.5)	0.16
≥61	18 959 (18.1)	31 162 (9.5)	0.25
Live birth parity			
Median (IQR)	1 (1-2)	1 (1-1)	0.06
1	77 911 (74.4)	253 138 (77.0)	0.06
≥2	26 752 (25.6)	75 774 (23.0)	0.06
Rural residence	12 475 (11.9)	33 052 (10.0)	0.06
Immigrant status			
Nonimmigrant	82 169 (78.5)	266 316 (81.0)	0.06
Immigrant^c^	22 494 (21.5)	62 596 (19.0)	0.06
**Newborn, at the second birth**			
Sex			
Female	51 016 (48.7)	160 021 (48.7)	0.002
Male	53 647 (51.3)	168 891 (51.3)	0.002
Birth weight, g^d^			
Median (IQR)	3431 (3110-3760)	3460 (3148-3782)	0.06
250-1499	492 (0.5)	1347 (0.4)	0.009
1500-2499	3667 (3.5)	9106 (2.8)	0.04
2500-3999	87 143 (83.3)	274 375 (83.4)	0.004
≥4000	13 353 (12.8)	44 067 (13.4)	0.02
Gestational age at birth, median (IQR), wk	39 (38-40)	39 (38-40)	0.01
Preterm birth (24-36 wk gestation)	5688 (5.4)	16 009 (4.9)	0.03
Any congenital or chromosomal anomaly	3146 (3.0)	9595 (2.9)	0.005

Abbreviation: Q, quintile.

^a^ A standardized difference greater than 0.10 is considered clinically meaningful.

^b^ Total number of Johns Hopkins Adjusted Clinical Group (ACG) System Aggregated Diagnosis Groups (ADG) (excluding pregnancy diagnosis), 1 to 365 days prior to the second birth hospitalization.

^c^ Excludes refugee immigrants.

^d^ Birth weight categories approximate the definitions of very low birth weight (≤1499 g), low birth weight (≤2499 g), normal birth weight (2500-3999 g), and high birth weight (≥4000 g).

**Figure.**
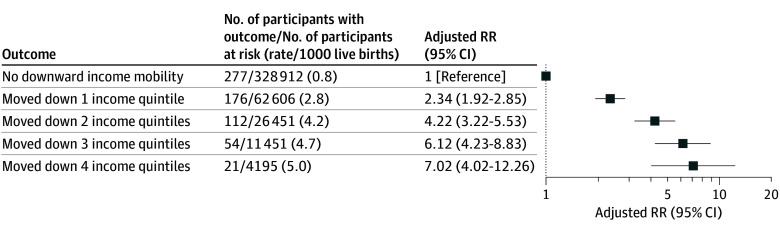
Risk of Newborn Discharge to Child Protective Services at the Second Birth Hospitalization Risk is presented by the degree in which a mother had downward neighborhood income mobility between 2 consecutive births in Ontario, Canada. In this model, absence of downward income mobility—the referent—was defined as residing in the same income quintile neighborhood at both births or moving to a higher-income quintile neighborhood between births. Relative risks (RRs) were adjusted for neighborhood income quintile at first birth hospitalization; age at second birth hospitalization; birth interval; parity; number of comorbidities within 1 to 365 days before the second birth hospitalization; residence at the second birth hospitalization; immigrant status; year of second birth hospitalization; and gestational age at birth for the second birth hospitalization.

## Discussion

In this study, a greater degree of maternal downward neighborhood income movement was associated with higher risk of newborn discharge to child protective services. As a limitation, some important maternal variables were unavailable, including individual-level income, race, and prior adverse life events. A woman may have had personal income loss or income gain without changing her neighborhood income Q between births. Likewise, a change in neighborhood income Q may not represent a change in personal income. Details about a newborn’s referral to child protective services or the duration of custody were also unavailable.

Identifying and mitigating modifiable factors coupled with maternal neighborhood income decline after a first birth may attenuate the need for future involvement of child protective services. Ongoing research should examine whether financial supplements can enhance neighborhood income stability and foster a stable maternal-newborn union.

## Notes

Supplement 1. **eTable 1.** Cohort Entry and Exclusion Criteria, Methods, and Coding to Identify Study Outcomes **eTable 2.** List of ICES Databases Used in the Current Study
eMethods.

eReferences.

Supplement 2.
Data Sharing Statement
