# Supplementary material for: Maternal Downward Neighborhood Income Mobility and Newborn Discharge to Child Protective Services
Source: JAMA Netw Open. 2024 Oct 23;7(10):e2440604. doi: 10.1001/jamanetworkopen.2024.40604 (PMC11581567; doi:10.1001/jamanetworkopen.2024.40604)
Supplement: Supplement 1. — eTable 1. Cohort Entry and Exclusion Criteria, Methods, and Coding to Identify Study Outcomes eTable 2. List of ICES Databases Used in the Current Study eMethods. eReferences. [file jamanetwopen-e2440604-s001.pdf]

Supplemental Online Content

Jairam JA, Cohen E, Diong C, et al. Maternal downward neighborhood income mobility and newborn discharge to child protective services. *JAMA Netw Open.* 2024;7(10):e2440604.
doi:10.1001/jamanetworkopen.2024.40604

eTable 1. Cohort Entry and Exclusion Criteria, Methods, and Coding to Identify Study Outcomes

eTable 2. List of ICES Databases Used in the Current Study

eMethods.

eReferences.

This supplemental material has been provided by the authors to give readers additional information about their work.

© 2024 Jairam JA et al. *JAMA Network Open.*

eTable 1. Cohort Entry and Exclusion Criteria, Methods, and Coding to Identify Study Outcomes

Assessment	Timing	Disease, procedure or condition	ICD-10-CA or CCI codes in CIHI-DAD, SDS and NACRS	Diagnostic & fee codes in OHIP	Other sources
Inclusion criteria	April 1, 2002 to March 31, 2018, at the time of the mother's index delivery hospitalization date, for the first and second births (selected) in the study cohort	• All hospital singleton livebirths and stillbirths at $20^{0/7}$ to $42^{0/7}$ weeks' gestation among women who were initially residing in an income quintile (Q) Q2, Q3, Q4, or Q5 area in Ontario at the time of their first birth during the study period, and who had a second consecutive birth also in Ontario.<br>• Among mothers with more than two births in the study period, an earlier livebirth or stillbirth was randomly selected to serve as the *first birth* in the cohort. A consecutive livebirth served as the *second birth*.<br>• Births limited to women aged 15 to 50 years old with a valid OHIP/IKN number from MOMBABY for the first and second index delivery hospitalizations.<br>• Stillbirth: fetal death arising *in utero*, or a newborn with no signs of life at birth, at ≥ 20 weeks' gestation -- among livebirths and stillbirths.	MOMBABY	--	RPDB
	Same as above	• Mother's postal code will be used to derive neighbourhood income Q.	--	--	RPDB, PCCF+, Statistic Canada census data
***All birth records*: Related exclusion criteria**	Same	• Records with warning for mother's IKN or KEY	MOMBABY/RPBD: warning for IKN/KEY (WARN not =”N”(No Warning)). Include N=no warning	--	--
	At the mother's index delivery hospitalization for the first birth	• Multiple births	MOMBABY (M_MULTIBIRTH='T' or B_MULTIBIRTH='T')	--	--

© 2024 Jairam JA et al. *JAMA Network Open.*

Assessment	Timing	Disease, procedure or condition	ICD-10-CA or CCI codes in CIHI-DAD, SDS and NACRS	Diagnostic & fee codes in OHIP	Other sources
	At the infant's index birth hospitalization for the first birth	• Infant gestational age at birth is <20 or ≥43 weeks' gestation or missing	MOMBABY:<br>a. B_GESTWKS_DEL not in MOMBABY<br>b. gestational age <20 or ≥43 weeks' gestation	--	--
	At the mother's index delivery hospitalization for the first birth	• Women without a second consecutive birth during study period	MOMBABY	--	--
	Same	• Women who had an invalid OHIP number or hospital number	MOMBABY/RPDB:<br>Invalid M_IKN (maternal IKN)<br>a. VALIKN ne 'V'<br>b. M_IKN not in RPDB (according to no sex and no bdate)<br>c. M_IKN with sex ='M' in RPDB	--	--
	At the time of arrival to Canada	• Immigrants with a landing date prior to their birth date	RPDB	--	IRCC-PRD
	Same	• Refugees and other immigrants			
***First selected livebirth or stillbirth during the study Period:* Related exclusion criteria**	At the mother's index delivery hospitalization for the first birth	Women aged < 15 or > 50 years, or age missing	MOMBABY/RPDB:<br>M_IKN age < 15 or > 50 or missing	--	--
	Same	Women who were a non-Ontario resident	--	--	RPDB:<br>M_IKN non-Ontario resident
	Same	Women ineligible for OHIP	--	--	RPDB
	Same	Women residing in income Q1 neighbourhoods or missing an income Q	--	--	RPDB, PCCF+, Statistics Canada census data
	Same	Rural/urban residence missing	--	--	RPDB, PCCF+

© 2024 Jairam JA et al. *JAMA Network Open.*

Assessment	Timing	Disease, procedure or condition	ICD-10-CA or CCI codes in CIHI-DAD, SDS and NACRS	Diagnostic & fee codes in OHIP	Other sources
Second selected live birth during the study Period: Related exclusion criteria	At the mother's index delivery hospitalization for the second birth	Women aged <15 or >50 years, or age missing	MOMBABY/RPDB:<br>M_IKN age < 15 or > 50 or missing	--	--
	Same	Women who were a non-Ontario resident	--	--	RPDB:<br>M_IKN non-Ontario resident (substr (prcddablk, 1,2 ne '35')
	Same	Women missing an income Q	--	--	RPDB, PCCF+, Statistics Canada census data
	Same	Women ineligible for OHIP	--	--	RPDB
	270 days or less during the 365-day lookback period before the mother's hospitalization for the second birth	Eligible for OHIP	--	--	RPDB
	Same	Rural/urban residence missing	--	--	RPDB, PCCF+
	At the time of arrival to Canada & the mother's delivery hospitalization date for the second birth	Immigrants with a landing date after their admission date for the second birth	RPDB	--	IRCC-PRD
	At the mother's index delivery hospitalization for the first and second births	< 161 days between these dates	MOMBABY: B_BDATE	--	--

© 2024 Jairam JA et al. *JAMA Network Open.*

Assessment	Timing	Disease, procedure or condition	ICD-10-CA or CCI codes in CIHI-DAD, SDS and NACRS	Diagnostic & fee codes in OHIP	Other sources
Main study exposure	At the mother's index delivery hospitalization for the first and second births	Degree of downward neighbourhood income mobility between the first & second consecutive births:<br>i) Moved down one income Q<br>ii) Moved down two income Q<br>iii) Moved down three income Q<br>iv) Moved four income Q<br>v) No downward income mobility (referent):– remaining in the same income Q area (i.e., Q2, 3, 4, or 5) between the first & second consecutive births, or moving to a higher income Q area (i.e., Q3, 4, or 5) between the first & second consecutive births.	--	--	RPDB, PCCF+, Statistic Canada census data
Main infant outcome	At the infant's birth hospitalization discharge date (for the second birth) -- restricted to births in MOMBABY from April 2002 to March 2018	Newborn discharge to child protection services by the *second birth* hospitalization discharge date – among livebirths	CIHI-DAD: DISHCDISP='03'	--	--
Descriptive variables and covariates	At the mother's index delivery hospitalization for the first and second births	Neighbourhood income quintile (Q2, 3, 4 or 5) at the first birth<br><br>Neighbourhood income quintile (Q1, 2, 3, 4 or 5) at the second birth	--	--	RPDB, PCCF+, Statistic Canada census data
	At the mother's index delivery hospitalization for the second birth	Rural vs. urban residence	--	--	RPDB, PCCF+
	At the time of arrival to Canada	Immigrant status: non-refugee immigrants vs. non-immigrants. (An immigrant woman must have been born outside of Canada and then migrated to, and obtained permanent residency, in Ontario).	--	--	IRCC-PRD

© 2024 Jairam JA et al. *JAMA Network Open.*

Assessment	Timing	Disease, procedure or condition	ICD-10-CA or CCI codes in CIHI-DAD, SDS and NACRS	Diagnostic & fee codes in OHIP	Other sources
	At the mother's delivery hospitalization for the second birth	Parity: number of previous livebirths (Previous term deliveries + Previous preterm deliveries), operationalized as a binary variable: 2 or more livebirths vs. 1 livebirth.	CIHI-DAD/MOMBABY	--	--
	At the infant's birth hospitalization for the second birth	Year of birth: 2003, 2004, 2005, 2006, 2007, 2008, 2009, 2010, 2011, 2012, 2013, 2014, 2015, 2016, 2017, 2018.	MOMBABY	--	--
	Same	Infant's biological sex assigned at birth	MOMBABY: b_sex	--	--
	Between 0 days (at the infant's birth hospitalization [for the second birth]) & up to the hospitalization discharge date	(3) Any congenital or chromosomal anomaly – among livebirths	ICD-10 Q00-Q99	--	--
	At the infant's birth hospitalization for the second birth	Preterm birth < 37 weeks' gestation – among livebirths	MOMBABY:B_GESTWKS_DEL	--	--
	At the mother's index delivery hospitalization for the second birth	Maternal age: 16-24, 25-29, 30-50	MOMBABY: M_AGE	--	--
	The infant's birth admission dates, for first and second births	Interpregnancy birth interval: Time (months) elapsed between infant's birth admission, for first and the second births. Calculated by subtracting the infant's birth admission date for the first birth from the infant's birth admission date for the second birth. Categories (months): 6-17, 18-60, or ≥ 61 months	MOMBABY: B_BDATE	--	--
	1 to 365 days prior to the hospitalization for the second birth	Number of comorbidities: Total number of Aggregated Diagnosis Groups (ADGs), excluding any pregnancy defined ADG.	ADGs are obtained from diagnosis codes in DAD, SDS and NACRS using The Johns Hopkins ACG® System Version 10 software	--	--

© 2024 Jairam JA et al. *JAMA Network Open.*

Assessment	Timing	Disease, procedure or condition	ICD-10-CA or CCI codes in CIHI-DAD, SDS and NACRS	Diagnostic & fee codes in OHIP	Other sources
	At the infant's birth hospitalization for the second birth	Birthweight (grams): 250-1499, 1500-2499, 2500-3999, ≥ 4000	MOMBABY	--	--
	Same	Gestational age at birth (weeks)	MOMBABY	--	--

Abbreviations: ACG: Adjusted Clinical Group; ADG: Aggregated Diagnosis Group; CIHI: Canadian Institute for Health Information; DAD: Discharge Abstract Database; CCI: Canadian Classification of Health Interventions; ICD-10-CA: International Classification of Diseases, 10th Revision, Canada; IRCC-PRD: Immigration, Refugees, and Citizenship Canada Permanent Resident Database; NACRS: National Ambulatory Care Reporting System; OHIP: Ontario Health Insurance Plan; PCCF+: Postal code conversion file plus (Statistics Canada); RPDB: Registered Persons Database; SDS: Same Day Surgery Database.

© 2024 Jairam JA et al. *JAMA Network Open.*

eTable 2. List of ICES Databases Used in the Current Study

Dataset name	Description
Aggregated Diagnosis Groups (ADG)	The Johns Hopkins Adjusted Clinical Groups (ACGs)® system assigns an ICD code to one of 32 diagnosis clusters known as Aggregated Diagnosis Groups (ADG). Individual diseases or conditions are placed into a single ADG based on 5 clinical dimensions: duration of the condition; severity of the condition; diagnostic certainty; etiology of the condition; and specialty care involvement. ICD codes within the same ADG are similar in both clinical criteria and expected need for healthcare resource. Individuals may have multiple diagnoses and belong to multiple ADGs (between zero and 32 ADGs).
Canadian Institute for Health Information Discharge Abstract Database (CIHI-DAD)	Captures all in-patient hospital admission records including obstetric deliveries and deaths. Diagnostic codes are based on the *International Statistical Classification of Diseases and Related Health Problems, Tenth Revision, Canada* (*ICD-10-CA*), and procedural codes are based on the *Canadian Classification of Health Interventions (CCI)*.
Immigration, Refugees and Citizenship Canada Permanent Residents Database (IRCC-PRD)	Captures demographic information on all international migrants who obtained permanent residency in Canada from January 1985 to March 2023.
Linked Delivering Mothers and Newborns (MOMBABY)	Derived from CIHI-DAD, provides linked inpatient hospital admission records of mothers and their infants.
National Ambulatory Care Reporting System (NACRS)	Collects data on hospital-and community-based ambulatory care, such as day surgery, outpatient and community-based clinics and emergency departments.
Ontario Health Insurance Plan Claims Database (OHIP)	Contains information about inpatient and ambulatory visits, consultations and procedures provided to Ontario residents eligible for Ontario's publicly funded health insurance system by fee-for-service health care practitioners (e.g., physicians, optometrists, laboratories for diagnostic tests etc.).
Postal Code Conversion File Plus (PCCF+)	A digital file that links the Canada Post Corporation (CPC) six-character postal code and Statistics Canada's standard geographic areas (e.g., dissemination area). Area-level income quintiles range from Q1 (lowest) to Q5 (highest) income neighbourhoods.
Registered Persons Database (RPDB)	Includes vital status and sociodemographic information about all individuals who have ever received an Ontario Health Insurance Plan (OHIP) number (e.g., date of birth, sex, and postal code).
Same Day Surgery (SDS)	Contain demographic, diagnostic, procedural and treatment information about all day surgical procedures.
Statistic Canada Census	Information from the Canadian Census, statistical information about the population including population counts and various levels of geography (e.g., census metropolitan areas, communities, census tracts etc.)

© 2024 Jairam JA et al. *JAMA Network Open.*

eMethods.

Study Design, Settings, and Participants

This population-based cohort study used multiple linked administrative databases from Ontario, Canada's most populous and ethnically diverse province. All permanent residents are eligible for universal, publicly funded health care, including antenatal and neonatal care. The cohort comprised all females who had two or more consecutive singleton births within hospital at 20 to 42 weeks' gestation, between April 1, 2002 and March 31, 2018. Among mothers with more than two births in the study period, an earlier livebirth or stillbirth from 20 to 42 weeks' gestation was randomly selected to serve as the *first birth* in the cohort. The next livebirth from 24 to 42 weeks' gestation served as the *second birth*. The study was limited to those residing in an income quintile (Q) Q2, 3, 4, or 5 (highest) neighbourhood at the time of the first birth, aged 15 to 50 years, and who had a valid Ontario Health Insurance Plan (OHIP) number. Mothers residing in a Q1 area at the first birth were excluded herein, since they could not experience downward neighbourhood income mobility below Q1, and the health of neonates of mothers residing in an income Q1 area has been described elsewhere.[1]

Data were linked using unique identifiers, held and analyzed at ICES. Data use was authorized under section 45 of Ontario's *Personal Health Information Protection Act*, and exempt from a research ethics board review. This study followed the STROBE reporting guideline.

Data Sources

This study used administrative data housed at ICES, an independent, not-for-profit research institute that has an inventory of Ontario's linkable health-related datasets. Ontario's health information privacy law allows ICES to collect and analyze health care and demographic data without consent for health system evaluation and improvement.

Datasets used for this study are valid and reliable sources for conducting perinatal research, as detailed in eTable 2.[2-4] All hospital-based births were identified in the ICES MOMBABY database, which captures about 98% of all births in Ontario, and links mothers and newborns hospital births records. Deidentified data sets were linked using unique encoded identifiers and analyzed at ICES.

Study Exposures

The exposure, degree of downward neighbourhood income Q mobility between the first and second recorded births (herein, called degree of downward income mobility) was categorized as, downward movement by: i) one income Q, ii) two income Q, iii) three income Q, or iv) four income Q, each relative to v) no downward income mobility – either residing in the same neighbourhood income Q at both births, or moving to a higher income Q neighbourhood between births (the reference group).

© 2024 Jairam JA et al. *JAMA Network Open.*

Neighbourhood income characterizes the socioeconomic profile of a local geographic area, it is dynamic, and can change over the life-course.[5] Neighbourhood income Q was defined as the average income per single-person equivalent in a dissemination area, adjusted for household size, and obtained from census area-level income data.[6,7] Neighbourhood income Q was derived from the mother's residential postal code at the time of her first and second birth hospitalizations, as described in our prior work.[1] The six-character postal code reflects a standard geographic dissemination area (herein, called a "neighbourhood") composed of one or more neighbouring dissemination blocks, with a population of 400 to 700 persons.[7,8]

Study Outcomes

As described in the main text of the research letter.

Statistical Analysis

Mean values and proportions were contrasted between mothers who experienced any downward income mobility vs. those who did not, with a standardized difference > 0.10 considered clinically important.

Modified Poisson regression with a robust error variance was used to estimate relative risks (RR) and 95% confidence intervals (CIs) for newborn discharge to child protection services at the second birth hospitalization, comparing mothers by their degree of downward income mobility between two births vs. no downward income mobility between two births. Statistical models adjusted for the following maternal characteristics: neighbourhood income Q (Q2, Q3, Q4, or Q5) at the first birth hospitalization; age (16-24, 25-29, or ≥ 30 years) at the second birth hospitalization; interval between the first and second births (6-17, 18-60, or ≥ 61 months), livebirth parity (≥ 2 or 1) at the second birth hospitalization; residence (rural or urban) at the second birth hospitalization; immigrant status (immigrant or non-immigrant); year of the second birth hospitalization (2003, 2004, 2005, 2006, 2007, 2008, 2009, 2010, 2011, 2012, 2013, 2014, 2015, 2016, 2017, or 2018); gestational age at birth for the second birth hospitalization (24-37 weeks' or ≥38 weeks') and number of comorbidities within 1 to 365 days before the second birth hospitalization. The latter was derived using the Johns Hopkins Adjusted Clinical Group (ACG)® System (version 10 software), excluding pregnancy-related diagnoses, and categorized into Aggregated Diagnosis Groups (ADG) (≤ 2, 3-4, 5-6, or ≥ 7).[10]

Statistical analyses were conducted from March to August 2024. All analyses were performed using SAS version 9.4 (SAS Institute Inc). The magnitude of RR and precision of corresponding 95% CI indicated significant differences between exposure groups.

© 2024 Jairam JA et al. *JAMA Network Open.*

eReferences.

1. Jairam JA, Vigod SN, Siddiqi A, et al. Neighborhood Income Mobility and Risk of Neonatal and Maternal Morbidity. *JAMA Netw Open.* 2023;6(5):e2315301.
2. Wen SW, Liu S, Marcoux S, Fowler D. Uses and limitations of routine hospital admission/separation records for perinatal surveillance. *Chronic Dis Can.* 1997;18(3):113-119.
3. Joseph KS, Fahey J, Canadian Perinatal Surveillance S. Validation of perinatal data in the Discharge Abstract Database of the Canadian Institute for Health Information. *Chronic Dis Can.* 2009;29(3):96-100.
4. Juurlink D PC, Croxford R, Chong A, Austin P, Tu J, et al. . *Canadian Institute for Health Information Discharge Abstract Database: A Validation Study.* Toronto: ICES;2006.
5. Shavers VL. Measurement of socioeconomic status in health disparities research. *Journal of the National Medical Association.* 2007;99(9):1013-1023.
6. Canadian Institute for Health Information. *Trends in Income-Related Health Inequalities in Canada: Methodology Notes.* Ottawa, ON: CIHI;2015.
7. Statistics Canada. Postal Code^{OM} Conversion File Plus (PCCF+) Version 8A, Reference Guide. December 2022 Postal codes. https://guides.library.queensu.ca/ld.php?content_id=34898339.
8. Statistics Canada. Dissemination Area. In. Ottawa, ON: Statistics Canada; 2021.
9. Austin PC. Using the standardized difference to compare the prevalence of a binary variable between two groups in observational research. *Commun Stat Simul Comput.* 2009;38(6):1228-1234.
10. John Hopkins. ACG System version 10.0 Technical Reference Guide. https://www.hopkinsacg.org/document/acg-system-version-10-0-technical-reference-guide/.

© 2024 Jairam JA et al. *JAMA Network Open.*
