# Supplementary material for: Maternal Downward Neighborhood Income Mobility and Newborn Discharge to Child Protective Services
Source: JAMA Netw Open. 2024 Oct 23;7(10):e2440604. doi: 10.1001/jamanetworkopen.2024.40604 (PMC11581567; doi:10.1001/jamanetworkopen.2024.40604)
Supplement: Supplement 2. — Data Sharing Statement [file jamanetwopen-e2440604-s002.pdf]

Data Sharing Statement

Jairam. Maternal Downward Neighborhood Income Mobility and Newborn Discharge to Child Protective Services. *JAMA Netw Open*. Published October 23, 2024.
doi:10.1001/jamanetworkopen.2024.40604

Data
Data available: The dataset from this study is held securely in coded form at ICES. While legal data sharing agreements between ICES and data providers (e.g., healthcare organizations and government) prohibit ICES from making the dataset publicly available, access may be granted to those who meet pre-specified criteria for confidential access, available at http://www.ices.on.ca/use-ices-data/ (email: das@ices.on.ca). The full dataset creation plan and underlying analytic code are available from the authors upon request, understanding that the computer programs may rely upon coding templates or macros that are unique to ICES and are therefore either inaccessible or may require modification.

Additional Information
Explanation for why data not available: ICES policy
